# Genetic and Clinical Features in 24 Chinese Distal Hereditary Motor Neuropathy Families

**DOI:** 10.3389/fneur.2020.603003

**Published:** 2020-12-14

**Authors:** Yongzhi Xie, Zhiqiang Lin, Pukar Singh Pakhrin, Xiaobo Li, Binghao Wang, Lei Liu, Shunxiang Huang, Huadong Zhao, Wanqian Cao, Zhengmao Hu, Jifeng Guo, Lu Shen, Beisha Tang, Ruxu Zhang

**Affiliations:** ^1^Department of Neurology, The Third Xiangya Hospital, Central South University, Changsha, China; ^2^Center for Medical Genetics and Hunan Key Laboratory of Medical Genetics, School of Life Sciences, Central South University, Changsha, China; ^3^Department of Neurology, Xiangya Hospital, Central South University, Changsha, China

**Keywords:** distal hereditary motor neuropathy, genetic diagnosis, genetic distribution, clinical features, GARS, SORD

## Abstract

**Background and Objectives:** Distal hereditary motor neuropathy (dHMN) is a clinically and genetically heterogeneous group of inherited neuropathies. The objectives of this study were to report the clinical and genetic features of dHMN patients in a Chinese cohort.

**Aims and Methods:** We performed clinical assessments and whole-exome sequencing in 24 dHMN families from Mainland China. We conducted a retrospective analysis of the data and investigated the frequency and clinical features of patients with a confirmed mutation.

**Results:** Two novel heterozygous mutations in *GARS*, c.373G>C (p.E125Q) and c.1015G>A (p.G339R), were identified and corresponded to the typical dHMN-V phenotype. Together with families with *WARS, SORD, SIGMAR1*, and *HSPB1* mutations, 29.2% of families (7/24) acquired a definite genetic diagnosis. One novel heterozygous variant of uncertain significance, c.1834G>A (p.G612S) in *LRSAM1*, was identified in a patient with mild dHMN phenotype.

**Conclusion:** Our study expanded the mutation spectrum of *GARS* mutations and added evidence that *GARS* mutations are associated with both axonal Charcot-Marie-Tooth and dHMN phenotypes. Mutations in genes encoding aminoamide tRNA synthetase (ARS) might be a frequent cause of autosomal dominant-dHMN, and *SORD* mutation might account for a majority of autosomal recessive-dHMN cases. The relatively low genetic diagnosis yield indicated more causative dHMN genes need to be discovered.

## Introduction

Distal hereditary motor neuropathy (dHMN) is a clinically and genetically heterogeneous group of disorders. The most common feature of dHMN is its slowly progressive distal limb muscle weakness and atrophy, with minimal or no sensory involvement (1). dHMN was classified into seven clinical subgroups according to the mode of inheritance and phenotype (2). Autosomal dominant (AD) dHMN includes typical dHMN with childhood onset (dHMN-I), dHMN with adulthood onset (dHMN-II), dHMN with upper limb predominance (dHMN-V), and dHMN with vocal cord palsy (dHMN-VII). Autosomal recessive (AR) dHMN comprises of chronic dHMN (dHMN-III), chronic forms with diaphragmatic palsy (dHMN- IV), and spinal muscular atrophy with respiratory failure (dHMN-VI).

To date, more than 30 causative genes implicated in dHMN have been identified (http://neuromuscular.wustl.edu/) (1, 3). There is an overlap between some dHMN and Charcot-Marie-Tooth (CMT) causative genes, such as *HSPB1, IGHMBP2*, and *DYNC1H1*, suggesting a broad overlap of clinical phenotypes (3, 4). Additionally, dHMN is one of the most uncommon subtypes of inherited peripheral neuropathy; more knowledge on its clinical features and molecular analysis is helpful for us to have a better understanding of the disease.

In this study, we report two novel mutations in *GARS*, c.373G>C (p.E125Q) and c.1015G>A (p.G339R), and summarize the clinical features of seven dHMN families with a definite genetic diagnosis.

## Materials and Methods

### Patients

Twenty-four Chinese dHMN families were recruited from the Third Xiangya Hospital and Xiangya Hospital between 2012 and 2019, among which two families with *WARS* or *SIGMAR1* mutation have been described previously (5, 6). All patients underwent complete neurological examination by two neurologists. The clinical criteria was consistent with patients presenting with a pure motor neuropathy with no sensory changes on electrophysiology (2). Routine hematological, biochemical, and electrophysiologic examinations were carried out in all probands and some patients. Disease severity was evaluated with CMT examination score (CMTES) (7). This study was approved by the ethical committee of the Third Xiangya Hospital of Central South University. Written informed consent was obtained from all participants.

### Molecular Genetic Analysis

Blood samples were obtained from all participants and genomic DNA was isolated using the standard Phenol-Chloroform method. Whole-exome sequencing (WES) was applied in 22 probands and trio-based WES was performed in two families. Low-quality reads and non-coding/synonymous variants were first filtered out. Exonic and splicing variants with minor allele frequency (MAF) of more than 0.005 in the context of genotype (heterozygous MAF>0.001; homozygous MAF>0.005) in gnomAD, 1000 Genome Project, dbSNP144 databases, and our in-house database were excluded. The novel variants, absent in all population databases and ClinVar, were made a priority for further analysis. *In silico* analysis was performed by using Mutation Taster, SIFT, and PolyPhen-2. The co-segregation analysis was performed through the application of Sanger sequencing and the short tandem repeat (STR) analysis was performed to confirm the parental origin of *de novo* variants. All variants were interpreted according to the American College of Medical Genetics and Genomics' (ACMG) standards and guidelines (8). Structural modeling analysis of novel p.E125Q and p.G339R mutations in human glycyl-tRNA synthetase (hGlyRS) were performed using PyMOL (The PyMOL Molecular Graphics System, Version 2.2 Schrödinger, LLC.).

## Results

### Genetic Findings and Analysis

**Figure 1:**
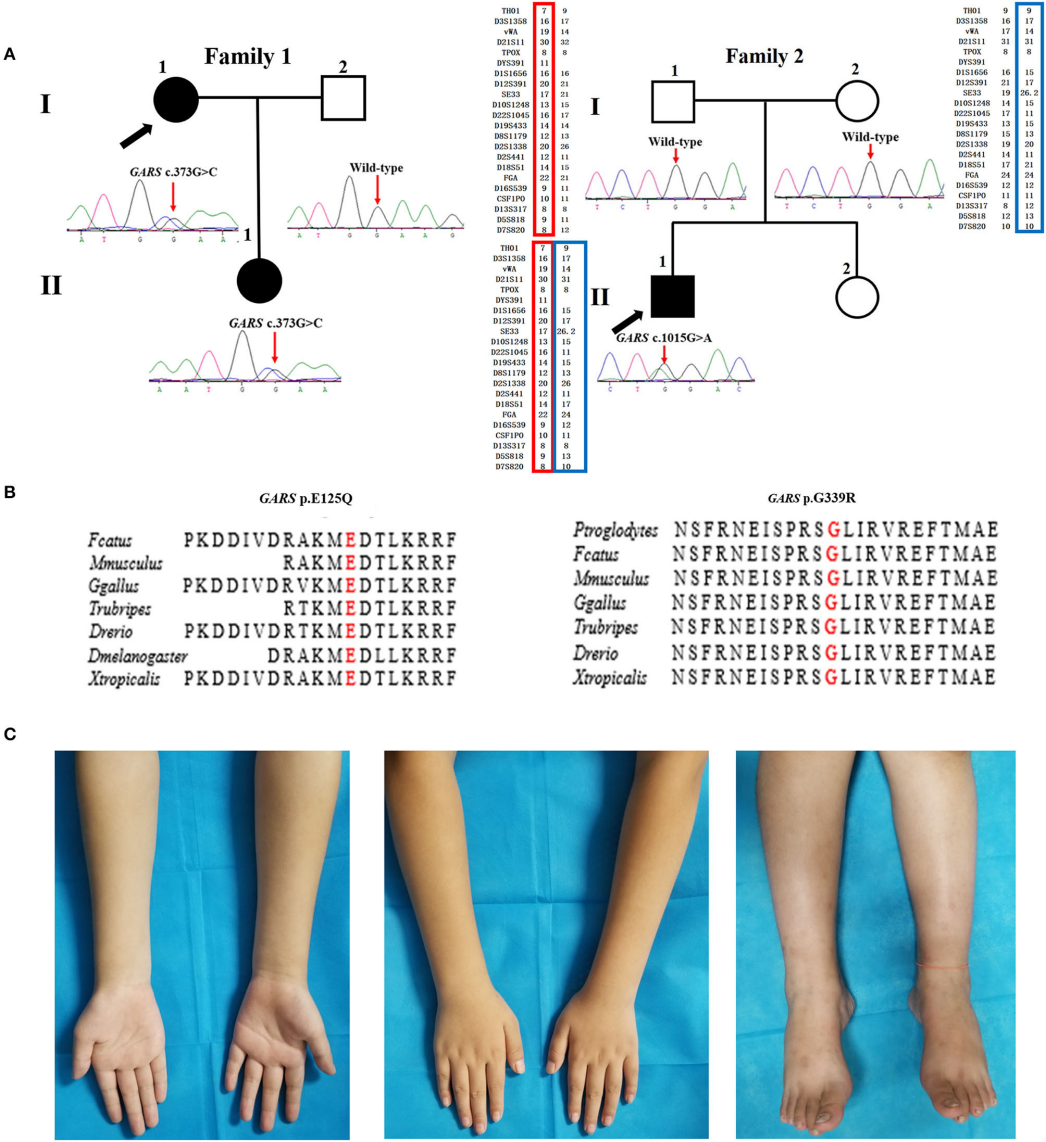
Pedigree, sequencing electropherograms, conservation analysis, and clinical imaging features of distal hereditary motor neuropathy (dHMN) families with *GARS* mutation. **(A)** Pedigrees of family1 (*GARS* c. 373G>C) and family 2 (*GARS* c.1015G>A), and chromatograms of the mutation sites confirmed by Sanger sequencing. The *GARS* c.1015G>A variant is shown to be a *de novo* variant by comparing 22 core STR markers. Square, male; Circle, female; Black filled symbol, clinically and electromyogram confirmed affected individual; Empty symbol, clinically healthy individual; M, mutant type; W, wild-type; Arrows, probands. **(B)** Conservation of the residues surrounding Glu acid (E) 125 (red) and Gly (G) 339 in *GARS* among species. **(C)** Images of the upper and lower limbs of the proband in family 2(II-1).

**Figure 2:**
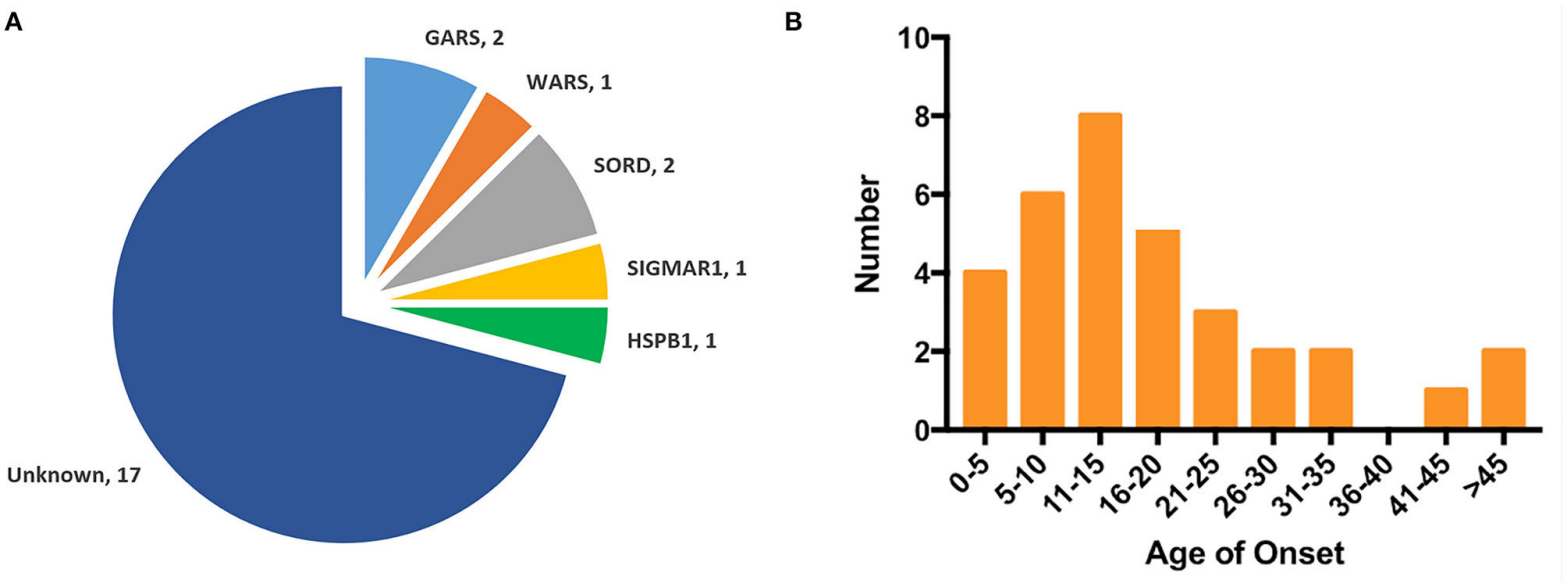
Genotype distribution and age of onset distribution in Chinese dHMN families. **(A)** Of the 24 dHMN families, the diagnosis was genetically confirmed in seven and a total of six genotypes have been identified. **(B)** Of the total 33 dHMN patients in 24 families, 75.8% (25/33) reported disease onset in the first two decades.

There were six AD families (25%), five AR families (20.8%), and 13 sporadic cases (51.2%) among the dHMN families. The novel variants c.373G>C (p.E125Q) and c.1015G>A (p.G339R) in *GARS* were absent in gnomAD, 1000 G, dbSNP144, and HGMD and were predicted to be damaging by the MutationTaster, SIFT, and PolyPhen-2. They were well-conserved among different species and completely co-segregated with the phenotype (Figures 1A,B). We further confirmed the p.G339R variant is a *de novo* variant by comparing 22 core STR markers (Figure 1A). According to the ACMG standards and guidelines, the variant c.373G>C (p.E125Q) was classified as likely pathogenic (PM2, PM5, PP1, PP3, PP4) and the variant c.1015G>A (p.G339R) as pathogenic (PS2, PM1, PM2, PP3, PP4). Together with five families harboring the reported c.941A>G (p.D341G) in *WARS*, c.757delG (p.A253Qfs^*^27) in *SORD*, c.151+1G>T (p.31_50del) in *SIGMAR1*, and c.539C>T (p.T180I) in *HSPB1*, the genetic diagnosis rate was 29.2% (7/24) in our Chinese dHMN families (Figure 2A). In addition, a total of 15 variants of uncertain significance (VUS) were identified, among which the variant c.1834G>A (p.G612S) in CMT2 causative gene *LRSAM1* was also absent in population databases and was predicted to be pathogenic by *in silico* analysis. However, we could not complete the co-segregation analysis because of deceased parents. Details are summarized in Supplementary Table 1.

### The Clinical Features of 7 dHMN Families With Definite Genetic Diagnosis

The mean age of onset was 17.7 ± 12.8 years (ranged from 1 to 50 years) in our dHMN patients, and 75.8% (24/33) of them reported disease onset in the first two decades (Figure 2B).

The proband harboring c.373G>C mutation in *GARS* (II:1 in F1) had difficulties in straightening their fingers at age 11, and subsequently developed difficulty in going up stairs and getting up after falls. During the high school period, she developed high-arched feet and atrophy of interosseous muscles and calves. Physical examinations revealed distal wasting and weakness in upper and lower limbs (score 3/5 in distal upper limbs and score 4/5 in distal lower limbs), normal sensation of all modalities, reduced bicep reflexes, knee reflexes, and ankle reflexes, stepping gait, and *pes cavus*. The median amplitudes of compound muscle action potentials (CMAPs) were significantly decreased (0.2 mV) with motor nerve conduction velocities (MNCVs) slightly reduced (37.5 m/s). The CMTES was 5. Her daughter (II:1), harboring the same mutation, developed difficulty in buttoning at age 9. Physical examinations at age 11 revealed mild muscle weakness restricted to the distal upper limbs (score 5-/5). No muscle atrophy, sensory disturbance, or other neurological deficits were observed. Electrophysiological studies revealed axonal polyneuropathy with decreased amplitudes of CMAPs (1.4 mV) and reduced MNCV (34.3 m/s). The CMTES was 1.

The sporadic patient carrying *de novo* c.1015G>A mutation in *GARS* (II:1 in F2) complained of weakness in upper limbs with writing difficulty at age 9. The weakness was gradually progressive with no involvement of the lower limbs when first examined at age 11. Physical examinations revealed distal wasting and weakness in the upper limbs (score 4/5 in distal upper limbs) and reduced bicep reflexes. The muscle strength in the lower limbs, sensation of all modalities, and tendon reflexes in the lower limbs were normal (Figure 1C). Electrophysiological examinations showed moderately decreased amplitudes of cMAPs (1.9 mV) with slightly reduced MNCV (37.2 m/s). His CMTES was 2.

Five other dHMN families also received genetic diagnosis, and their pedigrees are shown in Supplementary Figure 1. In brief, patients with c.941A>G mutation in *WARS* (F3) presented with juvenile to adult onset and mild to moderate disease severity (6). The proband with c.539C>T mutation in *HSPB1* (F4) presented with a childhood onset and typical dHMN-I phenotype. Two sporadic cases harboring homozygous c.757delG mutation in *SORD* (F5 and F6) presented with childhood disease onset and a mild phenotype. The homozygous c.151+1G>T mutation in *SIGMAR1* (F7) was associated with progressive neuropathy and pyramidal signs (5). The detailed clinical features of seven dHMN families are summarized in Table 1.

**Table 1:** Clinical features of patients in seven families harboring confirmed mutation.

**Family**	**Nucleotide**	**Inherit**	**Patients**	**Age at**	**Initial**	**Muscular atrophy^a^**	**Muscle weakness^b^**	**Reflexes^c^**	**Steppage**	***Pes***	**Median**	**Median**	**CMTES**
	**change**			**onset/exam (years)**	**symptom**	**(UL/LL)**	**(UL/LL)**	**(UL/LL)**	**gait**	***cavus***	**MNCV/CMAP**	**SNCV/SNAP**	
F1	*GARS* c.373G>C	AD	I:1 (proband)	11/36	Difficulty in straightening fingers	+/+	++/+	+/+	Yes	Yes	37.5/0.2	61.9/32	5
			II:1	9/11	Mild difficulty with buttoning	–/–	+/–	+/++	No	No	34.3/1.4	49.1/13	1
F2	*GARS* c.1015G>A	*De novo*	I:1	9/9	Writing difficulty	+/–	+/–	+/++	Yes	No	37.2/1.9	65.7/24.7	2
F3	*WARS* c.941A>G	AD	II:3 (proband)	18/51	Steppage gait	++/+++	++/+++	+/–	Yes	Yes	18.9/0.1	45.7/14	9
			I:1	23/79	Steppage gait	++/+++	++/+++	+/–	Yes	Yes	ND	ND	10
			II:1	17/53	Steppage gait	++/+++	++/+++	++/–	Yes	Yes	ND	ND	9
			III:1	15/18	Steppage gait	+/++	++/++	++/–	Yes	No	ND	ND	8
			III:2	16/19	Steppage gait	+/++	++/++	++/–	Yes	No	26.8/0.3	63.2/15.3	6
F4	*HSPB1* c.539C>T	AD	II:1 (proband)	13/29	Difficulty running	+/++	++/++	+/+	Yes	Yes	41/4.8	46.3/13	6
F5	*SORD* c.757delG	AR	II:1	10/19	Foot drop	++/++	+/++	++/–	Yes	Yes	56.4/2.6	60.7/11	5
F6	*SORD* c.757delG	AR	II:1	12/13	Difficulty walking	–/–	–/+	+/+	No	No	52.8/5.3	57.4/15	1
F7	*SIGMAR1* c.151+1G>T	AR	IV:1 (proband)	10/30	Foot drop, pes varus	++/+++	++/++	++/+++	Yes	Yes	43.4/1.2	58/45	8
			IV:3	9/37	Foot drop, pes varus	+/++	+/++	++/+++	Yes	Yes	ND	ND	9
			IV:5	12/42	Foot drop, pes varus	+/++	+/++	++/+++	Yes	Yes	ND	ND	10

*AD, autosomal dominant; AR, autosomal recessive; UL, upper limbs; LL: lower limbs; MNCV, motor nerve conduction velocity (in m/s); CMAP, compound muscle action potential (in mV); SNCV, sensory nerve conduction velocity (in m/s); SNAP, sensory nerve action potential (in uV); CMTES: Charcot-Marie-Tooth disease examination score; ND: not done*.

a *Patients with muscular atrophy; –, normal; +, mild; ++, moderate; +++, severe (involved in proximal muscle)*.

b *Patients with muscle weakness: –, normal; +, ≥4 in distal muscles on MRC (Medical Research Council) scale; ++, <4 in distal muscles on MRC scale; +++, proximal weakness (knee flexion and extension, elbow flexion and extension or above)*.

c *Tendon reflexes: –, loss; +, reduced; ++, normal; +++, hyperreflexia*.

### Molecular Structural Model Analysis of Novel Mutations p.E125Q and p.Q339R in GARS

**Figure 3:**
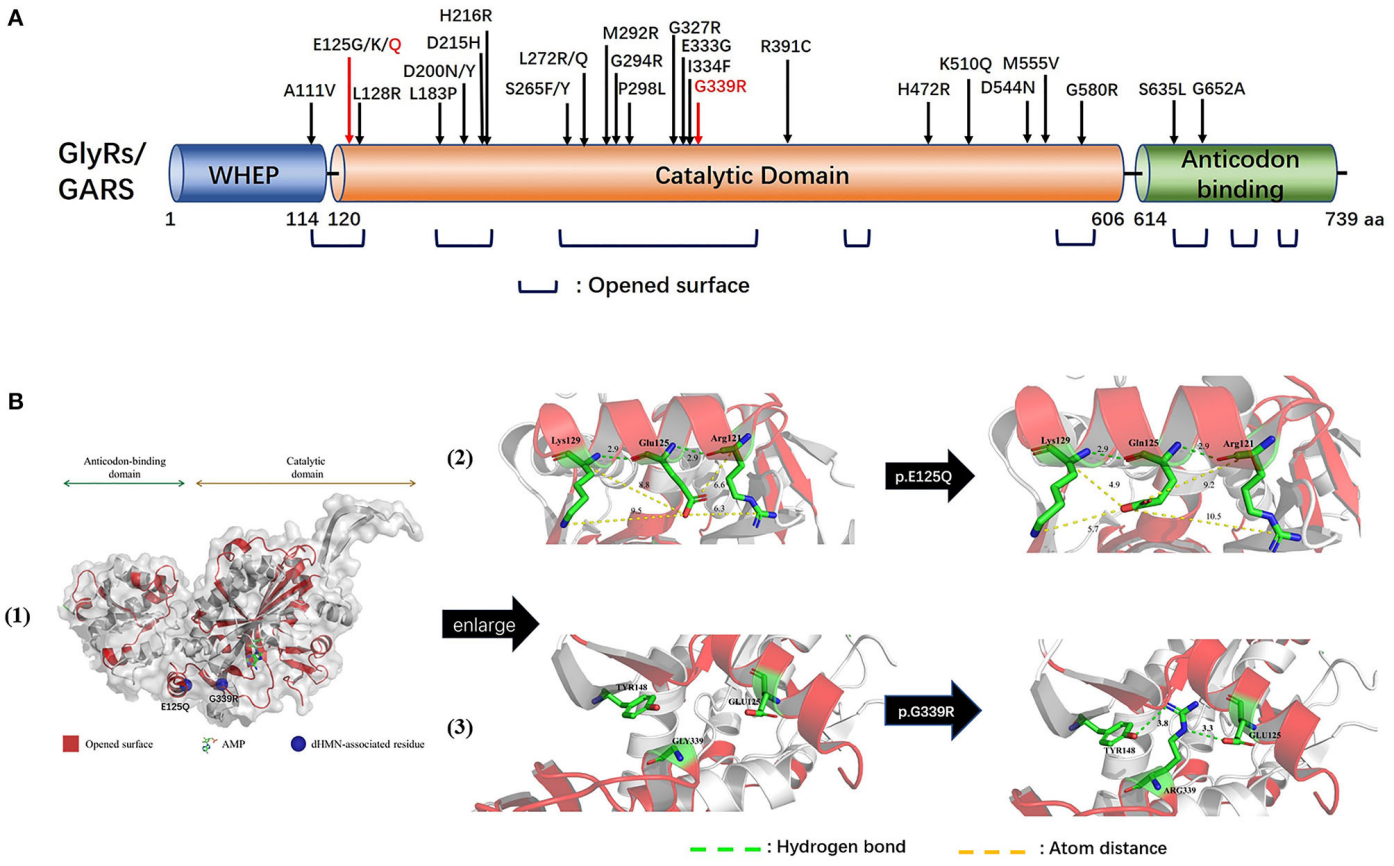
Schematic representation of GARS gene/protein and location of mutations. **(A)** Distribution CMT2D/dHMN-associated mutations in the three domains of the cytosolic human glycyl-tRNA synthetase (GlyRS). Red: Novel mutations identified with dHMN-V in this study. Most reported *GARS* mutations are located in the catalytic domain or lie in one of the eight opened surface regions. Amino acid nomenclature based on transcript NM 002047.2(739 a.a). **(B)** Molecular structures of integral type (left), wild type (middle), and mutant type (right) of human GlyRS (PDB ID: 4KR2). Both residues, p.E125 and p.G339, are strictly conserved in opened-up surface area of hGlyRS, among which p.G339 also located within the glycine-binding pocket. The substitution of p.E125Q altered the charge and space resistance with adjacent amino acids. The substitution of p.G339R engendered neomorphic hydrogen bonding interactions between residue p.G339 and p.Y148/p.E125.

Human glycyl-tRNA synthetase (hGlyRS) (Protein Data Bank, PDB, 4KR2) protein is composed of three domains: the WHEP domain, the evolutionarily conserved catalytic domain, and the anticodon binding domain, among which the catalytic domain is necessary for the catalytic activity of synthetase. Eight opened-up surface areas along hGlyRS had also been described as associating with structure conformational opening and dimer interface interaction. The novel mutations p.E125Q and p.Q339R were located at the catalytic domain and opened-up surface area (Figures 3A,B-1).

The p.E125Q substitution altered charge and space resistance with adjacent amino acids, which might affect the dimerization of the synthetase (Figure 3B-2). The p.G339R substitution engendered new hydrogen bonding interactions between p.G339 and p.Y148/ p.E125, which as described above are located in the opened-up surface area. The aberrant polar association among them might engender the formation of unexpected protein-protein interactions (Figure 3B-3). p.G339 is located within the glycine-binding pocket (9), and p.G339R may disturb the AMP active site which is essential for the aminoacylation activity of hGlyRS.

## Discussion

The genetic diagnosis rates and genetic distributions of pure dHMN are variable among different studies. Liu et al. reported 39% (16/41) of patients receive a genetic diagnosis in North China and *HSPB1, IGHMBP2*, and *GARS* were the most common causative genes (10), while our study showed a lower diagnostic rate (29.2%, 7/24) in South China and the most common causative genes were *GARS* and *SORD*. The genetic diagnosis rates reported by three studies from England were 14.3% (3/21), 26.4% (24/91), and 32.5% (13/40), respectively (4, 11, 12). Moreover, *DYNC1H1, GARS*, and *HSPB1* were found as the most leading disease-causing genes in a study conducted by Antoniadi et al., while *IGHMBP2, SYT2*, and *GARS* were more prevalent in a study conducted by Bansagi et al. (4, 11). Considering pure dHMN only accounted for a small proportion of inherited peripheral neuropathy, the small number of enrolled patients and sampling bias may contribute to these differences. More population-based studies are needed which could provide insights into the influence of geographical distribution. The overall molecular diagnosis rate is relatively low, indicating the presence of more causative genes that are yet to be identified and which would be helpful to interpret the pathogenesis of dHMN.

We detected two novel *GARS* mutations, c.373G>C (p.E125Q) and c.1015G>A (p.G339R), in two families who presented with a childhood disease onset with predominant upper limb involvement typical of a dHMN-V phenotype. The CMTES of affected members scored 1–5, indicating a mild phenotype. It is worth noting that CMTES is validated for CMT patients with both motor and sensory disturbances, and lack of sensory involvements in dHMN patients will result in lower CMTES scores. So far, 27 mutations in *GARS* have been linked to CMT2D/dHMN (Figure 3A), primarily associating with adolescent or early adult-onset disease with predominant upper limb involvement. Moreover, p.L272Q and p.E333D presented with predominant lower limb involvement, and p.D200Y and p.L272Q/R were associated with respiratory failure, indicating the clinical heterogeneity of *GARS* mutation (Supplementary Table 2). Our study supported that predominant upper limb involvement was the main phenotypic characteristic of *GARS* mutation. Of interest, p.E125G and p.E125K had been reported along with p.E125Q, indicating that p.E125 might be the hotspot site (11, 13–15). Our structural model analysis showed that p.E125Q might engender the formation of aberrant protein-protein interactions, consistent with a reported p.E125G mutation which did not affect aminoacylation activity but could compete with vascular endothelial growth factor for neuropilin 1 (16, 17). Further, p.G339R altered the polar association and might impair the dimerization of the synthetase of hGlyRS. Together with evidence that protein null alleles in mice do not cause dominant phenotypes (18), gain-of-function mechanism might be the main cause of *GARS*-related neuropathy. Suppression of the mutant allele (c.894_905del) of *GARS* by AAV9-mediated RNAi completely rescued the neuropathy in CMT2D-disease mice when treated at birth (19). Personalized therapies that promote allele-specific knockdown of mutant mRNA could be of therapeutic benefit for *GARS* related CMT2/dHMN.

Aminoacyl-tRNA synthetases (ARSs) have been implicated in inherited diseases. Five of the 20 genes encoding cytosolic ARSs (*GARS, AARS, WARS, YARS*, and *HARS*) were firmly linked to CMT2/dHMN, which is referred to as ARS-related CMT2/dHMN (20). Moreover, gain-of-function may be the shared mode of pathogenesis of ARS-related CMT2/dHMN because structural change of ARS rather than reduced enzyme function links to peripheral neuropathy (20–22). In our study, three families possessed mutations in ARS-related genes (*GARS* and *WARS*), accounting for 42.9% (3/7) of known confirmed families in our study. Of note, *GARS* was one of the most common causative genes of dHMN in North China and England and *AARS* has also been reported (4, 10–12), suggesting that ARS-related dHMN might account for a large part of dHMN. Recently, Cortese et al. identified homozygous or compound heterozygous c.757delG variant in *SORD* from 38 CMT2/dHMN families, suggesting a mutation in *SORD* as the most frequent cause of the recessive form of hereditary neuropathy (23). Here, we detected the homozygous c.757delG mutation in *SORD* in two sporadic cases with childhood disease onset and mild phenotype. *SORD* mutation might be the most common cause of Chinese AR-dHMN, requiring a larger population-based study to support this view. The novel c.1834G>A VUS in *LRSAM1* was in close proximity to the RING finger domain, where most dominant mutations have been reported. *LRSAM1* might be the same as some CMT2 genes, such as *GARS* and *HSPB1*, associated with the dHMN phenotype; however, more genetic evidence is needed to verify this.

In summary, we report two novel pathogenic mutations in *GARS:* c.373G>C (p.E125Q) and c.1015G>A (p.G339R). ARS-related dHMN might be a common AD-dHMN subtype, and *SORD* might be a frequent cause of AR-dHMN. The low genetic diagnosis yield indicates that there are more causative dHMN genes to be discovered.

## Data Availability Statement

The original contributions presented in the study are included in the article/Supplementary Materials, further inquiries can be directed to the corresponding author/s.

## Ethics Statement

The studies involving human participants were reviewed and approved by The ethical committee of the Third Xiangya Hospital of Central South University. Written informed consent to participate in this study was provided by the participants' legal guardian/next of kin. Written informed consent was obtained from the individual(s), and minor(s)' legal guardian/next of kin, for the publication of any potentially identifiable images or data included in this article.

## Author Contributions

RZ designed the study. YX, ZL, XL, LL, SH, HZ, BW, WC, ZH, JG, LS, and BT contributed patient material and clinical data. ZL ascertain the families and interpreted the genetic data. YX provide the first draft of the manuscript. PP edited the language. RZ revised the manuscript.

## Conflict of Interest

The authors declare that the research was conducted in the absence of any commercial or financial relationships that could be construed as a potential conflict of interest.

## Supplementary Material

Supplementary Figure 1 The pedigrees of dHMN families with reported mutations. Square: male; Circle: female; Black filled symbol: clinically and electromyogram confirmed affected individual; Empty symbol: clinically healthy individual; M: mutant type; W: wild-type; Arrows: probands.

Supplementary Table 1 Bioinformatics analysis and ACMG classification of the variants detected by whole-exome sequencing.

Supplementary Table 2 Clinical and molecular features of mutations in the *GARS* gene.
