# Supplementary figures and images for: Genetic and Clinical Features in 24 Chinese Distal Hereditary Motor Neuropathy Families
Source: Front Neurol. 2020 Dec 14;11:603003. doi: 10.3389/fneur.2020.603003 (PMC7767876; doi:10.3389/fneur.2020.603003)

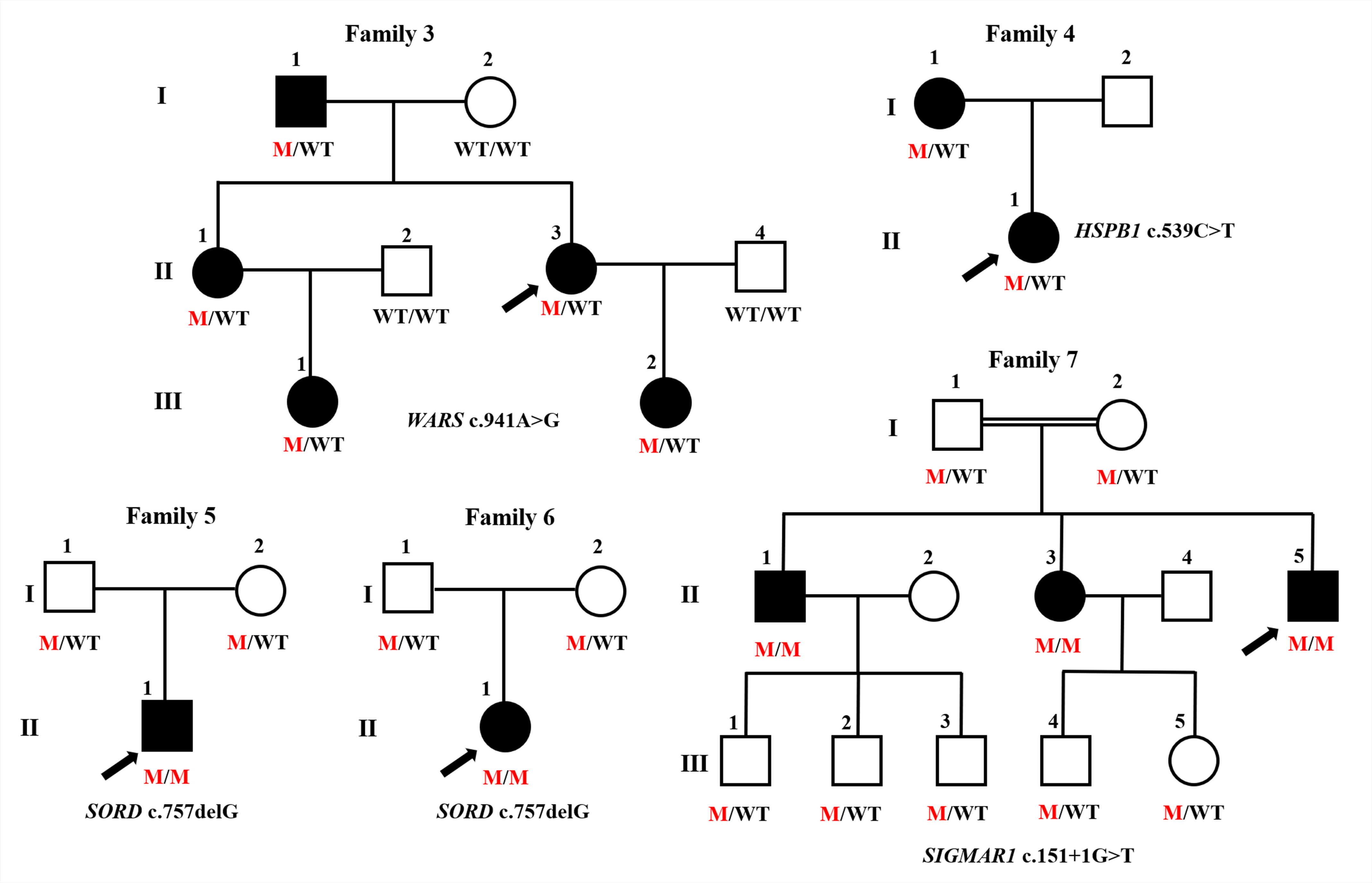

Supplement: Supplementary Figure 1 — The pedigrees of dHMN families with reported mutations. Square: male; Circle: female; Black filled symbol: clinically and electromyogram confirmed affected individual; Empty symbol: clinically healthy individual; M: mutant type; W: wild-type; Arrows: probands. [file Image_1.TIF]
