# Supplementary material for: Genetic and Clinical Features in 24 Chinese Distal Hereditary Motor Neuropathy Families
Source: Front Neurol. 2020 Dec 14;11:603003. doi: 10.3389/fneur.2020.603003 (PMC7767876; doi:10.3389/fneur.2020.603003)
Supplement: Supplementary Table 1 — Bioinformatics analysis and ACMG classification of the variants detected by whole-exome sequencing. [file Data_Sheet_1.docx]

Supplementary Material

**Population databases**

**Genome Aggregation Database (gnomAD): http://gnomad.broadinstitute.org/**

**1000 Genomes Project database (1000G): http://www.1000genomes.org/**

**Single Nucleotide Polymorphism Database (dbSNP): http://www.ncbi.nlm.nih.gov/snp/**

**ClinVar: http://www.ncbi.nlm.nih.gov/clinvar/**

***In silico* prediction**

**Mutation Taster: http://mutationtaster.org/**

**PolyPhen-2: http://genetics.bwh.harvard.edu/pph2/**

**SIFT-2: http://sift.jcvi.org/**

**sTable 1. Bioinformatics analysis and ACMG classification of the** **variants detected by whole-exome sequencing**

| **Family** | **Gene** | **Nucleotide changes** | **Amino acid**  **changes** | **Zygosity** | **Population databases** | | | | ***In silico* analysis** | | | **ACMG classification** |
| --- | --- | --- | --- | --- | --- | --- | --- | --- | --- | --- | --- | --- |
|  |  |  |  |  | **gnomAD** | **1000G** | **dbSNP** | **ClinVar** | **Mutation**  **taster** | **Polyphen2** | **SIFT** |  |
| 1 | *GARS* | c.373G>C | p. E125Q | Heterozygote | NF | NF | NF | NF | D | D | D | Likely Pathogenic (PM2, PM5, PP1, PP3, PP4) |
| 2 | *GARS* | c.1015G>A | p. G339R | Heterozygote | NF | NF | NF | NF | D | D | D | Pathogenic (PS2, PM1, PM2, PP3, PP4) |
| 3 | *WARS* | c.941A>G | p. D341G | Heterozygote | NF | NF | NF | Yes | D | D | D | Pathogenic (reported) |
| 4 | *HSPB1* | c.539C>T | p. T180I | Heterozygote | NF | NF | NF | Yes | D | T | B | Pathogenic (reported) |
| 5 | *SORD* | c.757delG | p. A253Qfs*27 | Homozygote | 0.0041 | NF | NF | Yes | D | D | D | Pathogenic (reported) |
| 6 | *SORD* | c.757delG | p. A253Qfs*27 | Homozygote | 0.0041 | NF | NF | Yes | D | D | D | Pathogenic (reported) |
| 7 | *SIGMAR1* | c. 151+1G>T | p.31_50del | Homozygote | NF | NF | NF | Yes | N/A | N/A | N/A | Pathogenic (reported) |
| 8 | *LRSAM1* | c.1834G>A | p. G612S | Heterozygote | NF | NF | NF | NF | D | D | D | VUS ( PM1, PM2, PP3) |
| 9 | *SIGMAR1* | c.176C>G | p.S59C | Heterozygous | 8.69e-06 | NF | rs769182234 | NF | D | D | D | VUS |
| 10 | *TGM6* | c.1282G>T | p.A428S | Heterozygous | 2.44e-05 | 0.0002 | rs556958028 | NF | D | D | B | VUS |
| 11 | *UBQLN2* | c.1718C>T | p.P573L | Heterozygous | 5.60e-06 | NF | NF | NF | D | D | T | VUS |
| 12 | *SH3TC2* | c.283C>G | p.L95V | Heterozygous | 2.03e-05 | 0.0002 | rs541695222 | NF | D | D | D | VUS |
| 13 | *MME* | c.2236A>C | p.K746Q | Heterozygous | 4.07E-06 | NF | NF | NF | D | T | B | VUS |
| 14 | *WARS* | c.1327G>T | p.A443S | Heterozygous | 1.64e-05 | 0.0004 | rs139914390 | NF | D | T | B | VUS |
| 15 | *AP4E1* | c.3010A>C | p.T1004P | Heterozygous | NF | NF | NF | NF | D | T | B | VUS |
| 16 | *VRK1* | c.83T>G | p.I28R | Heterozygous | 4.07e-06 | NF | rs778633021 | NF | D | T | D | VUS |
|  | *SLC5A7* | c.1214A>C | p.K405T | Heterozygous | 2.60e-04 | 0.0006 | rs199693962 | NF | D | D | B | VUS |
| 17 | *RTN2* | c.374G>A | p.G125D | Heterozygous | 1.02e-04 | NF | rs776994539 | NF | D | T | D | VUS |
| 19 | *PLEKHG5* | c.2012T>G | p.L671W | Heterozygous | NF | NF | NF | NF | D | D | D | VUS |
| 20 | *IFRD1* | c.342G>A | p.M114I | Heterozygous | 1.219e-05 | 0.0002 | rs562479630 | NF | D | T | B | VUS |
| 21 | *VPS37A* | c.1168C>T | p.H390Y | Heterozygous | 0.0002 | 0.0008 | rs200490159 | NF | D | D | D | VUS |
| 24 | *MYH14* | c.1039G>A | p.G347S | Heterozygous | 5.693e-05 | NF | rs775868764 | NF | D | T | B | VUS |

gnomAD: The Genome Aggregation database; 1000G: 1000 genomes project; dbSNP: The Single Nucleotide Polymorphism Database; NF: Not found; N/A: Not appreciable; D: Disease-causing/Damaging; B: Benign; T: Tolerable; ACMG: The American College of Medical Genetics and Genomics; VUS: Variants of uncertain significance.

**sTable 2 Clinical and molecular features of mutations in the *GARS* gene**

| **Mutations** | **Domains** | **Inheritance** | **Diagnosis** | **Age at onset** | **Distribution** | **Other Phenotypes** | **References** |
| --- | --- | --- | --- | --- | --- | --- | --- |
| p. R111V | RNA-binding | S | dHMN | 12 y | UL>LL | - | Rohkamm et al. |
| p. E125Q | - | AD | dHMN | 12 y | UL>LL | - | This study |
| p. E125K | - | S | dHMN | 3 mon | UL>LL | - | Natalie et al. |
| p. E125G | - | AD | CMT2D/dHMN | 18 y | UL>LL  (dHMN) | - | Antonellis et al  Sivakumar et al. |
| p. L128R | Catalytic core | AD | dHMN | 10 y | UL>LL | - | Yu et al. |
| p. L183P | Catalytic core | AD | dHMN | 21 y | UL>LL | - | Antonellis et al  Sivakumar et al |
| p. D200Y | Catalytic core | S | CMT2D | 3 mon | UL=LL | Respiratory failure | Liao et al. |
| p. D200N | - | AD | dHMN | 15 y | UL>LL | Increased deep tendon reflexes | Lee et al. |
| p. D215H | - | AD | CMT2D | 14-20 y | UL=LL | - | Nan et al. |
| p. H216R | Catalytic core | Unknown | dHMN | Unknown | UL>LL | - | Natalie et al |
| p. S265F | Catalytic core | AD | dHMN | 13 y | LL=UL | - | Lee et al. |
| p. S265Y | Catalytic core | S | CMT2D | 12 y | UL>LL | Epilepsy | Yalcouyé et al. |
| p. L272R | Catalytic core | S | dHMN | 7-10 mon | UL=LL | Respiratory failure | Chung et al. |
| p. L272Q | - | S | CMT2D | 7 y | LL>UL | Facial muscle atrophy and respiratory failure | Kawakami et al. |
| p. M292R | - | S | CMT2D | 2 y | UL=LL | wheelchair-bound | Liao et al. |
| p. G294R | - | AD | CMT2D | Unknown | UL>LL | - | Antonellis et al.  Sivakumar et al. |
| p. P298L | Catalytic core | Unknown | CMT2D | adolescence | UL>LL | - | Abe et al. |
| p. G327R | Catalytic core | Unknown | dHMN | Unknown | UL>LL | - | Natalie et al. |
| p. E333G | - | S | Infantile SMA | 1.6 y | - | - | Chae et al. |
| p. E333D | - | AD | CMT2D | 7 y | LL>UL | - | Sun et al. |
| p. I334F | Catalytic core | AD | dHMN | 11-18 y | UL=LL | - | James et al. |
| p. G339R | - | S | dHMN | 10 y | UL>LL | - | This study |
| p. R391C | - | S | CMT2D | 18 y | UL>LL | Brisk reflexes | Sivera et al. |
| p. H472R | - | - | dHMN | 26 y | - | - | Sivakumar et al. |
| p. K510Q | - | Unknown | dHMN | Unknown | UL>LL | - | Natalie et al. |
| p. D554N | - | AD | CMT2D/dHMN | 10-50 y | UL>LL | - | Del Bo et al. |
| P. M555V | - | Unknown | CMT2D | Unknown | UL>LL | - | Natalie et al. |
| p. G580R | - | AD | dHMN | 13-26 y | UL>LL | - | Antonellis et al.  Sivakumar et al. |
| p. S635L | Anticodon binding | AD | CMT2D | 4 y | LL>UL | - | James et al. |
| p. G652A | Anticodon binding | S | Infantile SMA | 6 mon | LL>UL | Weakness of eye closure | James et al. |

*GARS*: glycyl-tRNA synthetase, S: Sporadic, AD: autosome dominant, dHMN: Distal hereditary motor neuropathy, CMT: Charcot-Marie-Tooth disease, SMA: Spinal muscular atrophy, y: year, mon: month, UL: upper limbs, LL: lower limbs. Amino acid nomenclature based on transcript NM 002047.2(739 a.a).
